# TRDMT1 methyltransferase gene knockout attenuates STING-based cell death signaling during self-extracellular RNA-mediated response in drug-induced senescent osteosarcoma cells

**DOI:** 10.1007/s00018-025-05835-1

**Published:** 2025-08-13

**Authors:** Gabriela Betlej, Anna Deręgowska, Maciej Wnuk, Dominika Błoniarz, Tomasz Szmatoła, Katarzyna Klimczak, Jagoda Adamczyk-Grochala, Julia Świętoń, Anna Lewińska

**Affiliations:** 1 Faculty of Biotechnology, Collegium Medicum, University of Rzeszow, Pigonia 1, Rzeszow, 35-310 Poland; 2 Department of Basic Sciences, University of Agriculture in Krakow, Mickiewicza 24/28, Cracow, 30-059 Poland

**Keywords:** Self-extracellular RNA sensing, TRDMT1, STING, Drug-induced senescence, osteosarcoma

## Abstract

Under stress conditions, endogenous biomolecules such as nucleic acids or proteins can be released from damaged cells and considered as damage-associated molecular patterns (DAMPs) activating innate immune system and context-dependent responses. In the present study, self-extracellular RNA was obtained from dying (RNA D) and senescent (RNA S) cellular models of osteosarcoma (OS), characterized by NGS, and tested against proliferating and non-proliferating (etoposide-indued senescent) OS cells (U-2 OS, SaOS-2, MG-63, 143B). RNA D and RNA S induced apoptosis, nitro-oxidative stress, nucleic acid sensing pathways and cytokine production, and RNA m^5^C methyltransferase-based responses (TRDMT1 and NSUN2) in proliferating OS cells. In drug-induced senescent OS cells, *TRDMT1* gene knockout (KO) prevented STING activation, related proinflammatory response, and cell death. Furthermore, IFN-β binding RNA partners were identified, namely *NSUN2*, *NSUN5*, *NSUN6*, *CDKN1A*, *MYC*, and *RAD51* transcripts and these interactions were compromised in *TRDMT1* KO cells and upon RNA D and RNA S treatment. *TRDMT1* KO also resulted in replication stress in OS cells that was potentiated by RNA D and RNA S stimulation and associated with elevated levels of APOBEC3A and APOBEC3G, members of the cytidine deaminase protein family. In conclusion, we showed that *TRDMT1* KO restricted STING-based immune and cell death response to RNA D and RNA S in non-proliferating drug resistant OS cells that might have potential therapeutic implications.

**Supplementary Information:**

The online version contains supplementary material available at 10.1007/s00018-025-05835-1.

## Introduction

The innate immune system can recognize both microbe-specific molecules (pathogen-associated molecular patterns, PAMPs) and host-derived molecules (damage-associated molecular patterns, DAMPs) by means of membrane-bound and cytoplasmic pattern recognition receptors (PRRs) [1, 2]. DAMPs, released from stressed, damaged or dying cells, can be classified into five main molecular categories, namely nucleic acids, proteins, ions, glycans, and metabolites [1, 2]. In immune and non-immune cells (e.g., fibroblasts, epithelial and endothelial cells), DAMPs can be sensed, among others, by Toll-like receptors (TLRs, such as TLR2, 3, 4, 7, 9), C-type lectin receptors (CLRs, such as DNGR1), NOD-like receptors (NLRs, such as NLRP3), RIG-I-like receptors (RLRs, such as RIG-I, retinoic acid inducible gene I, and MDA5, melanoma differentiation-associated protein 5), and cytosolic DNA sensors (CDSs, such as cGAS, cyclic GMP-AMP synthase) [1–3]. In contrast to PAMPs, DAMPs can trigger sterile inflammation, a secretion of proinflammatory agents (cytokines, chemokines, etc.) and recruitment of immune cells without an infection [1, 2]. Sterile inflammation mediated by the activation of DAMP-sensing receptors may provoke context-dependent responses, namely both beneficial and detrimental effects such as the stimulation of tissue repair and regeneration, and antitumor immunity, and the development of cellular senescence, autoimmune diseases and cancer, respectively [1, 2, 4].

RLRs, such as RIG-I (recognition of dsRNA with > 20 bp) and MDA5 (recognition of dsRNA with > 1–2 kbp) RNA sensors, can discriminate between self and non-self-RNA as endogenous RNA can be protected against RLR signaling by RNA-binding proteins or adenosine deaminase acting on RNA 1 (ADAR1)-mediated modification (adenosine-to-inosine, A-to-I, RNA editing), respectively [1, 5]. If such protection is compromised, self-RNA can be considered as a DAMP activating RLR signaling and modulating, among others, cancer progression and therapeutic outcomes [6–8]. However, unprotected (unmasked) self-RNA may stimulate both cancer-promoting and cancer-inhibiting effects depending on cancer type and experimental settings [6–8]. For example, treatment with unshielded RN7SL1 RNA-containing exosomes resulted in RIG-I-based induction of tumor growth and anti-breast cancer therapy resistance [6, 7], whereas loss of ADAR1 sensitized mouse tumors to immunotherapy by MDA5-mediated stimulation of IFN-1 production and related proinflammatory response [8]. Furthermore, cancer cells of different origin were characterized by increased release of RNA (mainly rRNA) to the serum-free cell culture media compared to normal cells and this effect was potentiated under hypoxic conditions [9]. Extracellular RNA also promoted macrophage-associated secretion of TNF-α resulting in cancer invasion through endothelial barriers [9].

It is widely accepted that chemotherapeutics may potentiate the heterogeneity of cancer cells populations, e.g., chemotherapy-induced senescence may be activated as a side effect promoting proinflammatory tumor microenvironment and drug resistance [10]. As RNA can be also released from dying and drug-induced senescent cancer cells during chemotherapy, extracellular RNA-mediated responses and their consequences in cancer cells should be studied in more detail.

In the present study, four complementary in vitro cell models of osteosarcoma (OS), namely U-2 OS, SaOS-2, MG-63, and 143B cell lines [11] were used to investigate the effects of total RNA released from dying (RNA D) as well as etoposide-induced senescent OS cells (RNA S) on proliferating and non-proliferating OS cell populations with the emphasis of cell death signaling, nitro-oxidative stress, nucleic acid sensing pathways and RNA m^5^C methyltransferase-based responses. We show that TRDMT1 methyltransferase was involved in the response to RNA D and RNA S treatment in OS cells and *TRDMT1* gene knockout (KO) in etoposide-induced senescent OS cells diminished the activation of stimulator of interferon genes (STING), secretion of proinflammatory agents and related cell death signaling, thus promoting drug resistance.

## Materials and methods

### Osteosarcoma cells, culture conditions, and treatment protocols

Four cellular models of osteosarcoma (OS) in vitro were used, namely U-2 OS (92022711), SaOS-2 (89050205), MG-63 (86051601) (ECACC, Public Health England, Porton Down, Salisbury, UK), and 143B cell lines (CRL-8303™, ATCC, Manassas, VA, USA). Cells were typically seeded at the concentration of 10^4^ cells per cm^2^ and cultured in Dulbecco’s Modified Eagle Medium (DMEM) supplemented with 10% (v/v) fetal bovine serum (FBS) and antibiotic/antimycotic mix (100 U/ml penicillin, 0.1 mg/ml streptomycin, and 0.25 µg/ml amphotericin B) (Corning, Tewksbury, MA, USA) at 37 °C in the atmosphere of 5% CO_2_. OS cells were sub-cultured using a trypsin/EDTA solution (0.25%, Corning, Tewksbury, MA, USA).

To activate chemotherapy-induced senescence program, OS cells were treated with 1 µM etoposide (E1383, Merck KGaA, Darmstadt, Germany) for 24 h and then the drug was discarded and cells were left in the culture for additional 7 days with medium exchange every 48 h [12]. In the case of selected *TRDMT1* KO cells, lower concentrations of etoposide (0.25 µM for *TRDMT1* KO 143B cells and 0.5 µM for *TRDMT1* KO SaOS-2 cells) were used to stimulate drug-induced senescence as *TRDMT1* KO cells were more sensitive to etoposide treatment than unmodified OS cells.

To collect total RNA released from dying cells (RNA D), U-2 OS, SaOS-2, MG-63, and 143B cells were treated with 15, 10, 20, and 10 µM etoposide for 72 h, respectively. To collect total RNA released from senescent cells (RNA S), U-2 OS, SaOS-2, MG-63, and 143B cells were treated with 1, 1, 5 and 2 µM etoposide for 24 h, respectively, and left in the culture for additional 7 days. Cell culture medium was then collected, cellular debris were removed from cell culture medium by centrifugation (300x*g*, 5 min, RT) and RNA D and RNA S as total RNA were extracted from supernatants using acid phenol: chloroform: isoamyl alcohol (125:24:1, pH 4.5) solution according to the manufacturer’s instructions (AM9722, Thermo Fisher Scientific, Waltham, MA, USA). The purity and concentration of isolated total RNA were analyzed using NanoDrop™ 2000 UV-Vis spectrophotometer (Thermo Fisher Scientific, Waltham, MA, USA). Total RNA samples were kept at −86 °C until further use (for stimulation analysis).

OS cells were stimulated with RNA D and RNA S using a standard lipofection protocol (Lipofectamine Stem Transfection Reagent, STEM00015, Thermo Fisher Scientific, Waltham, MA, USA). The amounts of RNA and lipofection reagent were adjusted according to the cell culture plate used and the manufacturer’s instructions. Briefly, OS cells were cultured in 96-well plates (10^4^ cells per a well) overnight, and 50 ng of RNA D or RNA S was then added in a form of RNA-lipid complexes (0.3 µl of lipofection reagent per a well) for 24 h. If OS cells were cultured in 12-well plate and 6-well plates, 500 ng of RNA D or RNA S and 3 µl of lipofection reagent, and 1 µg of RNA D or RNA S and 6 µl of lipofection reagent were used, respectively.

### Next generation RNA sequencing (NGS) and bioinformatics analysis

For NGS, total RNA released to the cell culture medium during etoposide-induced cell death (RNA D) or etoposide-induced cellular senescence (RNA S) was pulled from five biological replicates. Each biological replicate consisted of three technical replicates. RNA D or RNA S was reverse-transcribed to cDNA using Transcriptor First Strand cDNA Synthesis Kit (04896866001, Roche, Basel, Switzerland) according to the manufacturer’s instructions. cDNA was then amplified using EmeraldAmp MAX HS PCR Master Mix (RR330A, Takara Bio Inc., Shiga, Japan) according to the manufacturer’s instructions. NGS was conducted as an outsource service (Genomed S.A., Warsaw, Poland) using NovaSeq 6000/MiSeq Sequencing System (Illumina, Inc., San Diego, CA, USA) with 2 × 150/2 × 300 bases mode and with NEBNext^®^ Ultra™ II Directional RNA Library Prep Kit for Illumina^®^ (NEB) libraries. The first stage of the analysis was to remove adapters using the Cutadapt program [13]. Filtering was carried out, as a standard, using the quality parameter q 25 and the minimum length of readings m 15. For these processed sequencing data, quality reports were generated using the FASTQC program (https://www.bioinformatics.babraham.ac.uk/projects/fastqc/). A mapping was then carried out using the Hisat2 program [14] using only uniquely mapped reads for further analysis. The reads were mapped to the reference genome of *Homo sapiens* (https://ftp.ncbi.nlm.nih.gov/genomes/refseq/vertebrate_mammalian/Homo_sapiens/latest_assembly_versions/GCF_000001405.40_GRCh38.p14/). The Hisat program was used with the option to prepare the RNA-strandness RF library. The number of read pairs mapped to individual genes was then counted using the HTseq program [15] according to the transcript strand (–stranded = reverse) using the union default method.

After obtaining the results in the form of count tables for each RNA D and RNA S samples from four OS cell lines used, genes with raw counts greater than 10 were selected for further analysis. First, the BioMart software [16] was used to classify the identified genes based on their annotation as protein-coding, pseudogenes, snoRNAs, miRNAs or lncRNAs. Subsequently, the upsetR software [17] was utilized to assess the overlap of identified genes among OS-derived samples with respect to RNA S and D. Finally, gene enrichment analysis was conducted using the standalone KOBAS software, incorporating three databases: Reactome, GO Pathways, and KEGG [18]. The pathways exhibiting the highest fold enrichment and the most significant adjusted *p* values were identified with respect to RNA S and D.

### Transfection

To obtain *TRDMT1* gene knockout (KO) in OS cells, CRISPR-based system was used as previously described [12]. Briefly, for lipofection, control double nickase plasmid (sc-437281), TRDMT1/DNMT2 double nickase plasmids (h, sc-402709-NIC, h2, sc-402709-NIC-2) (Santa Cruz Biotechnology, Dallas, TX, USA), and Lipofectamine Stem Transfection Reagent (STEM00015, Thermo Fisher Scientific, Waltham, MA, USA) were used according to the manufacturer’s instructions. *TRDMT1* KO cells were selected upon puromycin treatment (sc-108071, Santa Cruz Biotechnology, Dallas, TX, USA) and *TRDMT1* KO was verified using anti-TRDMT1/DNMT2 antibody (A-7, sc-271513, Santa Cruz Biotechnology, Dallas, TX, USA) and western blotting [12].

### RNA uptake

For RNA uptake analysis, RNA D or RNA S was labeled using ULYSIS™ Alexa Fluor™ 647 (650/670 nm) Nucleic Acid Labeling Kit according to the manufacturer’s instructions (U21660, Thermo Fisher Scientific, Waltham, MA, USA). OS cells were then stimulated with stained RNA, and stained RNA was tracked in the cells using Amnis^®^ FlowSight^®^ imaging flow cytometer and IDEAS software (version 6.2.187.0) (Luminex Corporation, Austin, TX, USA). Two parameters were analyzed, namely intensity_MC_Ch01 (bright field, BF) and intensity_MC_Ch11 (Cy5). Representative dot plots and microphotographs are shown.

### MTT test

Upon stimulation with RNA D or RNA S, metabolic activity of OS cells was investigated using MTT test [19]. The metabolic activity of untreated control cells was assumed as 100%. The effect of lipofection reagent (LF) was also studied.

### Apoptosis

Upon stimulation with RNA D or RNA S, apoptotic cell death was studied in proliferating and non-proliferating (etoposide-induced senescent) OS cells (WT and *TRDMT1* KO cells) using flow cytometry (Muse^®^ Cell Analyzer) and Muse^®^ Annexin V & Dead Cell Kit (Cytek Biosciences, Amsterdam, The Netherlands) [20]. Briefly, OS cells were stained according to the manufacturer’s instructions and four cell subpopulations were analyzed and quantified (%), namely live cells (negatively stained cells), early apoptotic cells with Annexin V positive staining, late apoptotic cells with Annexin V positive staining and 7-AAD positive staining, and necrotic cells with 7-AAD positive staining. Representative dot plots are shown.

### Oxidative stress and nitrosative stress

Upon stimulation with RNA D or RNA S, oxidative stress and nitrosative stress were analyzed as the levels of superoxide and nitric oxide, respectively. Superoxide levels were studied using flow cytometry (Muse^®^ Cell Analyzer) and Muse^®^ Oxidative Stress Kit containing dihydroethidium (Cytek Biosciences, Amsterdam, The Netherlands) [20]. OS cells were stained according to the manufacturer’s instructions and two subpopulations were revealed, namely superoxide-positive and superoxide-negative (%). Representative histograms are shown. Nitric oxide levels were investigated using flow cytometry (Muse^®^ Cell Analyzer) and Muse^®^ Nitric Oxide Kit containing DAX-J2™ Orange (Cytek Biosciences, Amsterdam, The Netherlands) [20]. OS cells were also stained using 7-AAD staining to discriminate between live and dead cells with increased nitric oxide production. Briefly, upon dedicated staining and cytometric evaluation, four cell subpopulations were documented, namely live cells negative for DAX-J2™ Orange staining and 7-AAD staining, live cells with increased nitric oxide levels (positive for DAX-J2™ Orange staining), dead cells with increased nitric oxide levels (positive for DAX-J2™ Orange staining and 7-AAD staining), and dead cells with unaffected nitric oxide levels (positive for 7-AAD staining) (%). Representative dot plots are shown.

### AKT and MAPK1/3 activity

Upon stimulation with RNA D or RNA S, OS cells were fixed, permeabilized, and immunostained using Muse^®^ PI3K/MAPK Dual Pathway Activation Kit containing a phospho-specific AKT (Ser473)-Alexa Fluor^®^555 conjugated antibody and a phospho-specific MAPK3 (former name ERK1)/MAPK1 (former name ERK2) (Thr202/Tyr204, Thr185/Tyr187)-PECy5 conjugated antibody (Cytek Biosciences, Amsterdam, The Netherlands) [20]. The activation of PI3K (AKT phosphorylation) and MAPK (MAPK1/3 phosphorylation) signaling pathways was then analyzed using flow cytometry and dedicated software (Muse^®^ Cell Analyzer, Cytek Biosciences, Amsterdam, The Netherlands). Four cell subpopulations were recognized, namely negative cells with no AKT and MAPK1/3 activation, cells with activated AKT (with phosphorylated AKT), cells with activated MAPK1/3 (with phosphorylated MAPK1/3), and cells with dual activation of AKT and MAPK1/3 (with phosphorylated AKT and phosphorylated MAPK1/3). Representative dot plots are shown.

### Imaging cytometry

Proliferating and non-proliferating (etoposide-induced senescent WT and *TRDMT1* KO) OS cells were stimulated with self-extracellular RNA (RNA D or RNA S), fixed, and immunostained [19] and the levels (or activity) of selected components of nuclei acid sensing pathways and RNA m^5^C methyltransferase-based response were studied. The following primary and secondary antibodies were used, namely anti-MDA5 (700360, 1:100), anti-RIG-I (700366, 1:100), anti-STING (MA5-26030, 1:100), anti-phospho-STING (Ser366) (PA5-105674, 1:100), anti-TLR3 (PA5-20183, 1:200), anti-TLR7 (PA1-28109, 1:200), anti-IFN-β (PA5-20390, 1:50), anti-NFκB (PA5-16545, 1:50), anti-IL-1β (P420B, 1:200), anti-IL-6 (TA500067, 1:100), anti-IL-8 (M801, 1:500), anti-DNMT2/TRDMT1 (sc-365001, 1:100), anti-NOP2/NSUN1 (PA5-59073, 1:600), anti-NSUN2 (702036, 1:250), anti-NSUN3 (PA5-57561, 1:200), anti-NSUN4 (720212, 1:250), anti-NSUN5 (PA5-54228, 1:200), anti-NSUN6 (PA5-61119, 1:600), anti-p21 (MA5-14949, 1:800), anti-APOBEC3A (PA5-99584, 1:100), anti-APOBEC3G (PA5-89318, 1:50), and secondary antibodies anti-rabbit conjugated to Texas Red (T2767, 1:1000), anti-mouse conjugated to Texas Red-X (T6390, 1:1000), anti-mouse conjugated to FITC (F2761, 1:1000), and anti-rabbit conjugated to FITC (F2765, 1:1000) (Thermo Fisher Scientific, Waltham, MA, USA, Santa Cruz Biotechnology, Dallas, TX, USA). Nuclei were stained using Hoechst 33342 staining. Fluorescence signals were captured and analyzed using IN Cell Analyzer 6500 HS and IN Carta software (Cytiva, Marlborough, MA, USA). The protein levels (total, cytosolic or nuclear) are presented as relative fluorescence units (RFU). STING activation was analyzed as a ratio of phosphorylated STING foci to STING cytosolic signals. TLR3 and TLR7 levels, in relative fluorescence units, were analyzed in endosomes using org intensity parameter. APOBEC3G levels were calculated as APOBEC3G foci per cell.

### DNA fiber assay

To assess self-extracellular RNA and *TRDMT1* KO-mediated changes in DNA replication dynamics in proliferating OS cells, RNA D and RNA S-stimulated WT and *TRDMT1* KO MG-63 cells were incubated with 100 µM 5-chloro-2’-deoxyuridine (CldU, C6891, Merck KGaA, Darmstadt, Germany) at room temperature (RT) for 30 min, washed three times with DPBS, and then incubated with 100 µM 5-iodo-2’-deoxyuridine (IdU, I7125, Merck KGaA, Darmstadt, Germany) at RT for 30 min. Cells were then washed three times with DPBS, harvested by trypsinization, washed once with DPBS, and embedded in agarose plugs according to the manufacturer’s instructions (EXT-001, Comb Helix DNA Extraction Kit, Genomic Vision, Bagneux, France). Genomic DNA was extracted by incubating plugs in lysis buffer at 50 °C overnight, then washed three times in wash buffer for 1 h and once at 4 °C overnight. Plugs were then melted at 68 °C for 20 min, at 42 °C for 10 min, and incubated at 42 °C overnight for agarose digestion. Subsequently, samples were combed onto coverslips (COV-001, Genomic Vision, Bagneux, France) using a Fiber Comb - Molecular Combing System (Genomic Vision, Bagneux, France) at RT. After combing, coverslips were desiccated at 65 °C for 2 h. Coverslips were denatured using denaturing buffer (a solution containing 0.5 M NaOH and 1 M NaCl) at RT for 5 min, washed three times with DPBS for 2 min, dehydrated in 70% and 100% ethanol for 5 min each, and then air dried for 30 min. Coverslips were blocked using 5% BSA in DPBS at 37 °C for 30 min, and then incubated with primary antibodies, namely anti-IdU (1:10, SAB3701448, Merck KGaA, Darmstadt, Germany), and anti-BrdU (1:40, ab6326, Abcam, Cambridge, UK) at 4 °C overnight. Coverslips were washed then three times with DPBS and incubated with secondary antibodies, namely anti-mouse conjugated to FITC (1:88, F2761) and anti-rat conjugated to Alexa Fluor^TM^555 (1:88, A-21434 (Thermo Fisher Scientific, Waltham, MA, USA) at RT for 1 h. Samples were then washed three times with DPBS and mounted with an antifade. Images were captured using an Olympus BX61 fluorescent microscope (Shinjuku, Japan) with objective 60x. Data were analyzed using ImageJ using the plugin RGB measure (IdU, green signals; CIdU, red signals) (https://imagej.net/ij/). Results are presented as a ratio of IdU to CldU normalized to control that reflects DNA replication dynamics [21].

### IFN-β-RNA interaction analysis

The catRAPID omics v2.1 with interaction prediction module between protein and human transcriptome (with module protein-coding RNA) [22] was employed to predict protein-RNA interactions of interferon beta (IFN-β). IFN-β sequence in FASTA format was downloaded from UniProt database (P01574 IFNB_HUMAN). catRAPID is a computational tool that assesses interaction propensities based on physico-chemical properties, including secondary structure, hydrogen bonding, and van der Waals forces [22]. To ensure high-confidence predictions, the results were analyzed by examining the interaction scores and Z-scores. Interaction scores above 50 were considered indicative of strong binding affinities. A Z-score greater than 1 was used as an additional layer of validation to minimize false positives. The results of predictions were then checked for gene enrichment analysis using three databases: Reactome, GO Pathways and KEGG using KOBAS standalone software [18]. The obtained pathways associated with cell cycle, DNA replication, RNA degradation, RNA methylation, regulation of RNA stability and with a *p* value < 0.05 were plotted in a form of a circos plot using R software and circlize package [23].

### RNA immunoprecipitation (RIP) assay

RIP assay was conducted using Magna RIP^®^ RNA-Binding Protein Immunoprecipitation Kit (17–700, Merck KGaA, Darmstadt, Germany). Briefly, WT and *TRDMT1* KO MG-63 cells were stimulated with RNA D or RNA S, and total cell lysates were then used for RIP with anti-IFN-β antibody (PA5-20390, Thermo Fisher Scientific, Waltham, MA, USA). The immunoprecipitated RNA was reverse-transcribed to cDNA using Transcriptor First Strand cDNA Synthesis Kit (04896866001, Roche, Basel, Switzerland), followed by RT-PCR amplification using Applied Biosystems StepOnePlus™ Real-Time PCR System, dedicated TaqMan Master Mix and sequence-specific primers: *NSUN2* (Hs00214829_m1), *NSUN5* (Hs00216128_m1), *NSUN6* (Hs01013871_m1), *CDKN1A* (Hs00355782_m1), *MYC* (Hs00153408_m1), *RAD51* (Hs00947967_m1), and *18S* (Hs99999901_s1) as a housekeeping gene (Thermo Fisher Scientific, Waltham, MA, USA). For binding analysis, the amount of selected transcript upon RIP protocol, namely sample incubation with dedicated anti-IFN-β antibody was calculated based on the difference between cycle threshold (Ct) values of WT CTR sample and *TRDMT1* KO CTR sample, or WT treated sample and *TRDMT1* KO CTR sample, or *TRDMT1* KO treated sample and *TRDMT1* KO CTR sample (ΔCt) and then using the formula 2^−ΔCt^. Thus, each sample for each transcript was normalized to *TRDMT1* KO CTR sample. Data are presented as a fold change of binding to IFN-β.

### Statistical analysis

The results are shown as mean ± standard deviation from at least three biological replicates. For data presentation of selected experiments, box and whisker plots with median, lowest, and highest values were also considered. Statistically significant differences between untreated control cells (CTR) and RNA-treated cells were evaluated using one-way analysis of variance (ANOVA) and Dunnett’s multiple comparison test. Statistically significant differences between WT cells and *TRDMT1* KO cells were evaluated using ANOVA and Tukey’s multiple comparison test. GraphPad Prism 8 software was used. *P* values lower than 0.05 were considered as statistically significant.

## Results and discussion

### Characterization of RNA released from dying and senescent OS cells using NGS

**Fig. 1:**
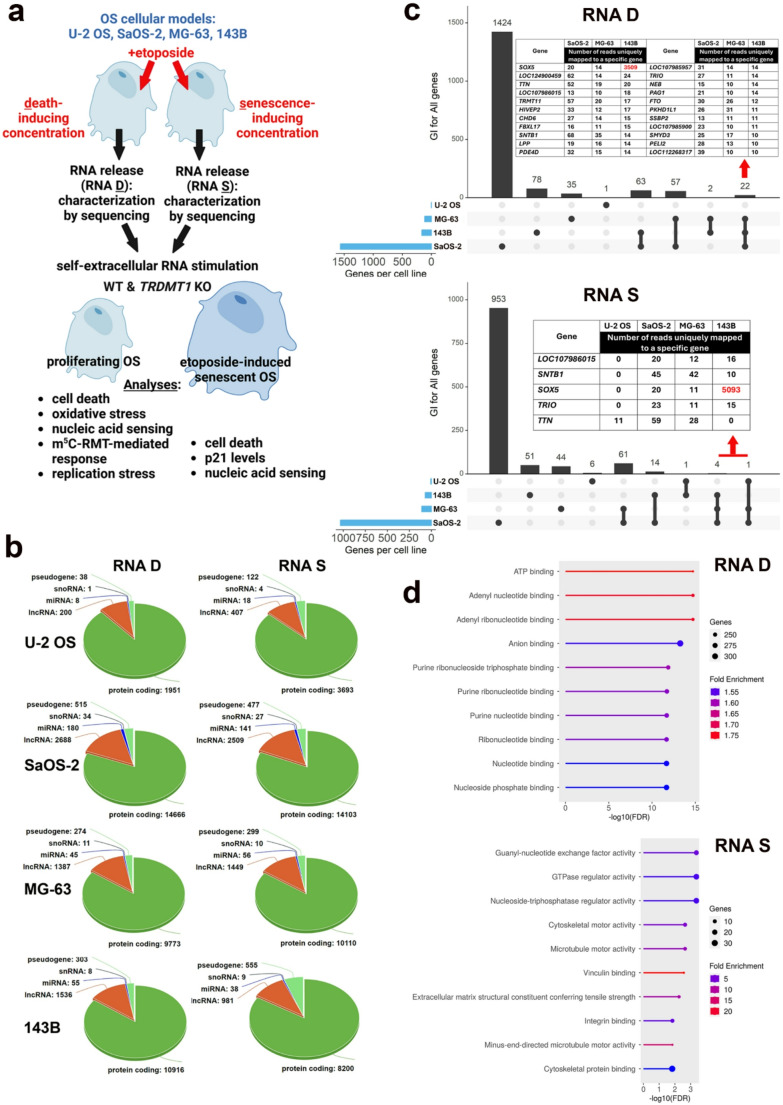
A scheme of study design (**a**) and NGS-based analysis of total RNA released from dying (RNA D) and drug-induced senescent (RNA S) osteosarcoma (OS) cells (**b-d**). **a** Four cellular models of OS in vitro were used, namely U-2 OS, SaOS-2, MG-63, and 143B cells. OS cells were treated with etoposide, DNA damaging chemotherapeutic agent, at both death and senescence-inducing concentrations and total RNA released to cell culture medium, RNA D and RNA S, respectively, was characterized using NGS. Proliferating and/or non-proliferating (drug-induced senescent) OS cells were then stimulated with RNA D and RNA S to reveal RNA-associated responses (cell death, oxidative stress, nucleic acid sensing, changes in m^5^C RMT (NSUNs and TRDMT1) levels, and replication stress). **b** For NGS, total RNA released to the cell culture medium during etoposide-induced cell death (RNA D) or etoposide-induced cellular senescence (RNA S) was pulled from five biological replicates. Each biological replicate consisted of three technical replicates. The total number within the main identified categories (protein coding genes, pseudogenes, snoRNA, miRNA, lncRNA) in RNA D and RNA S samples based on their annotation. Pie charts are shown; snoRNA, small nucleolar RNA; miRNA, microRNA; lncRNA, long non-coding RNA. More information can be found in the Supplementary Material. **c** The overlap of identified genes between RNA D (top) and RNA S (bottom) samples obtained from four OS cell lines. Set intersections in a matrix layout were revealed using the UpSet plot. The number of total, shared, and unique genes identified in RNA D (top) and RNA S (bottom) samples are shown. Blue bars in the y axis indicate the total number of identified genes in each RNA D (top) and RNA S (bottom) sample from a particular OS cell line. Black bars in the x axis indicate the number of genes shared across RNA D (top) and RNA S (bottom) samples from OS cell lines connected by the black dots in the body of the plot. Common genes are also denoted using red arrows and their number of reads is listed in a table (in a frame). The highest number of reads for *SOX5* gene is highlighted in red. More information can be found in the Supplementary Material.**d** The molecular pathways with the highest fold enrichment and the most significant adjusted *p* values were revealed using the standalone KOBAS software and three databases Reactome, GO Pathways, and KEGG. RNA D samples (top), RNA S samples (bottom). More information can be found in the Supplementary Material

It is widely accepted that chemotherapy may provoke different responses in targeted cancer cells and non-targeted neighboring cells (e.g., cell death, senescence, drug resistance), and treated cells may also communicate with unaffected cells by means of DAMPs modulating therapeutic effects [1, 2, 10, 24]. However, little is known if RNA liberated from drug-treated damaged cancer cells may affect the responsiveness of neighboring proliferating and non-proliferating (senescent) cancer cells. In the present study, we have tested the effects of RNA released form dying (RNA D) and senescent (RNA S) OS cells as a novel DAMP (alarmin) signal on proliferating and non-proliferating corresponding cells (Fig. 1a). For both in vitro systems - drug-induced cell death and drug-induced senescence (Fig. 1a), etoposide, one of anticancer drugs used to treat OS, was selected [25].

First, RNA D and RNA S were characterized using NGS (Fig. 1b-d). Four cellular models of OS were considered, namely U-2 OS, SaOS-2, MG-63, and 143B cells [11], and in all cases, NGS-based analysis revealed that the majority of released RNA was protein-coding RNA, from 81 to 89% for SaOS-2 and U-2 OS cells, respectively (Fig. 1b). The second most abundant fraction was long non-coding RNA (lncRNA), from 9 to 15% for U-2 OS and SaOS-2 cells, respectively (Fig. 1b). The fractions of microRNA and small nuclear RNA (snRNA) did not exceed 1% of total RNA analyzed (Fig. 1b). However, one should remember that our sequencing method is not compatible with small RNA sequencing and, thus, the analysis of small RNA pools in RNA D and RNA S samples might be underestimated. In the case of RNA S released from etoposide-induced senescent 143B cells, the fraction of pseudogenes reached 6% that differed from other RNA S samples obtained from other OS cells (from 2.5 to 2.9%) (Fig. 1b). However, the percentage composition of RNA D and RNA S was similar (Fig. 1b). We then analyzed the most abundant detected reads uniquely mapped to a specific gene in RNA D and RNA S released from four OS cell lines (Fig. 1c). The highest number of reads was noticed in RNA D and RNA S samples from SaOS-2 cells (Fig. 1c). According to applied cut-off value of ten and above for reads that uniquely mapped to a specific gene, we did not identify any common read that would be present in RNA D or RNA S originated from all four OS cell lines (Fig. 1c). However, 22 and 4 common reads were observed in RNA D and RNA S samples from SaOS-2, MG-63, and 143B cells, respectively (Fig. 1c). In RNA D and RNA S samples from 143B cells, unique reads for *SOX5* gene, a member of SRY-box transcription factor family with regulatory functions in development, cell-fate decision, and differentiation [26], was present in a number of more than 3500 and 5000, respectively, that was differed from other RNA D and RNA S samples (Fig. 1c, in red). SOX5, a regulator of chondrogenesis and neurogenesis [27, 28], was reported to be up-regulated in different types of cancer and associated with poor prognosis [26]. Perhaps elevated number of reads uniquely mapped to *SOX5* gene in RNA D and RNA samples from 143B cells may reflect more aggressive phenotype of 143B cells with metastatic potential compared to tumorigenic non-metastatic U-2 OS, SaOS-2, and MG-63 cells [11, 29]. In general, transcripts identified in RNA D and RNA S are involved in the regulation of nucleotide and ribonucleotide metabolism, and cytoskeletal function, respectively (Fig. 1d). According to our knowledge, there is no information on NGS-based precise composition of RNA released from dying or senescent cancer cells. It was reported that untreated cancer cells of different origin could liberate RNA into the serum-free cell culture medium in higher amounts compared to normal cells [9]. Furthermore, RNA release could be potentiated under hypoxic conditions [9]. Extracellular RNA was also subjected to agarose gel electrophoresis and the main component was rRNA with both 18S and 28S fractions [9]. As our NGS-based analysis of self-extracellular RNA did not include rRNA and focused mainly on protein-coding RNA, one cannot exclude that rRNA is also present in RNA D and RNA S samples and rRNA may take part in RNA-based response in treated OS cells.

### Self-extracellular RNA induces apoptosis and nitro-oxidative stress in OS cells

**Fig. 2:**
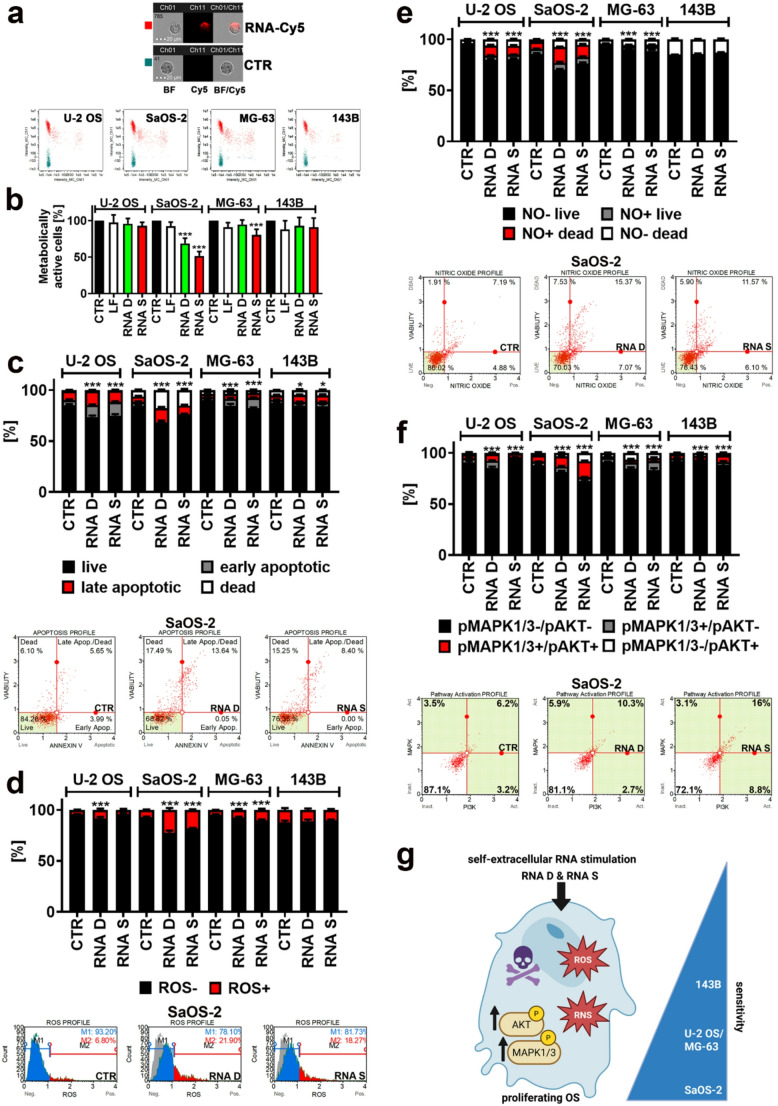
Self-extracellular RNA (RNA D and RNA S) uptake (**a**), RNA-mediated changes in metabolic activity (**b**), induction of apoptosis (**c**), oxidative stress (**d**), nitrosative stress (**e**), AKT and MAPK1/3 activity (**f**) in proliferating OS cells. **a** RNA uptake was tracked using labeled RNA and imaging flow cytometry. Representative microphotographs and dot plots are presented. Intracellular signals of labeled RNA were revealed using Cy5 channel (red). **b** Metabolic activity was studied using MTT assay. Metabolic activity at control untreated conditions was assumed as 100%. Lipofection control (LF) was also considered. **c** Apoptosis was studied using Annexin V staining and flow cytometry. Representative dot plots are also presented.**d** The levels of intracellular superoxide were analyzed using dihydroethidium and flow cytometry. Representative histograms are also presented; M1, superoxide-negative population (blue histogram); M2, superoxide-positive population (red histogram); CTR histogram is also denoted (gray histogram). **e** The levels of nitric oxide were investigated using DAX-J2^TM^ Orange and flow cytometry. Representative dot plots are also presented. **f** The activity of AKT and MAPK1/3 was analyzed using anti-phospho-AKT and anti-phospho-MAPK1/3 antibodies and flow cytometry. Representative dot plots are also presented. **b-f** Bars indicate SD, *n* = 3 (three biological replicates), ^***^p < 0.001, ^*^p< 0.05 compared to untreated control (CTR) (ANOVA and Dunnett’s a posteriori test). **g** A summarizing scheme showing the results of analyzed parameters (**b-f**) in OS cells. ROS, reactive oxygen species; RNS, reactive nitrogen species; RNA D, RNA released from dying OS cells; RNA S, RNA released from drug-induced senescent OS cells.

Second, the effects of RNA D and RNA S on cell viability and redox balance were studied in proliferating OS cells (Fig. 2). We did confirm that RNA in a form of RNA-lipid complexes could be uptaken by four OS cell lines (Fig. 2a). RNA D and RNA S did not affect the metabolic activity of U-2 OS and 143B cells as judged by MTT test (Fig. 2b). In contrast, RNA D and RNA S treatment resulted in a decrease in the metabolic activity of SaOS-2 cells, and RNA S treatment promoted a decrease in the metabolic activity of MG-63 cells (Fig. 2b). The inhibitory effects were the most pronounced in the case of RNA S-treated SaOS-2 cells (Fig. 2b). Lipofection reagent (LF) did not cause any changes in metabolic activity of four cell lines used and was excluded from further analysis (Fig. 2b). RNA D and RNA S induced apoptotic cell death in all four OS cell lines as documented by Annexin V staining (Fig. 2c). RNA D and RNA S also promoted necrotic cell death in SaOS-2 cells (Fig. 2c). The most sensitive to RNA D and RNA S treatment were SaOS-2 and U-2 OS cells (Fig. 2c). Except for 143B cells, RNA D and RNA S also stimulated superoxide production (oxidative stress) (Fig. 2d) and nitric oxide production (nitrosative stress) (Fig. 2e) in OS cells. SaOS-2 cells were the most susceptible to RNA D and RNA S-mediated redox imbalance compared to other RNA-treated OS cells (Fig. 2d and e). RNA D and RNA S also activated PI3K and MAPK pathways as judged by increased phosphorylation of AKT and MAPK1/3 kinases, respectively (Fig. 2f). The most accented activation of AKT and MAPK1/3 kinases was observed in RNA-treated SaOS-2 cells (Fig. 2f). The activation of PI3K and MAPK pathways is mainly considered as a pro-survival signal frequently deregulated in a number of cancer cell types [30, 31]. However, the effect of the activation of AKT or MAPK1/3 kinases on cancer cell fate might also depend on cellular context and promote cell death signaling pathways [31, 32]. It is widely accepted that increased production of reactive oxygen species (ROS) may stimulate the activation of PI3K/AKT and Ras/MAPK1/3 pathways in cancer cells [33]. In our experimental conditions, RNA-mediated nitro-oxidative stress was associated with increased activity of AKT or MAPK1/3 kinases and related cell death (Fig. 2). SaOS-2 cells were the most sensitive to RNA-mediated induction of apoptotic cell death, nitro-oxidative stress, and changes in the activity of PI3K and MAPK pathways among OS cell lines analyzed, whereas 143B cells were the least affected upon RNA treatment (Fig. 2g). Of course, such divergent response may rely on different genetic background and mutation status in four OS cell lines used. Furthermore, RNA D and RNA S samples from SaOS-2 cells were characterized by the highest number of identified genes among other RNA samples with the highest number of reads for *SNTB1*, *TTN*, *TRMT11*, *HIVEP2*, *CHD6*, *PDE4D*, *TRIO*, and *PELI2* genes, and some non-coding RNAs such as LOC124900459 in RNA D sample (Fig. 1c). However, the role of these genes during RNA-based response in SaOS-2 cells should be further verified. One should also remember that 143B cells have a metastatic potential, while U-2 OS, SaOS-2, and MG-63 cells are tumorigenic non-metastatic cell lines [11, 29]. Perhaps more aggressive metastatic OS cells, here 143B cells, are less susceptible to self-extracellular RNA stimulation than non-metastatic ones such as SaOS-2 cells. Treatment with self-extracellular RNA may also have more clinical relevance in primary OS tumors than metastases, but this assumption needs to be further experimentally verified.

### Self-extracellular RNA stimulates the activity of nucleic acid sensing pathways

**Fig. 3:**
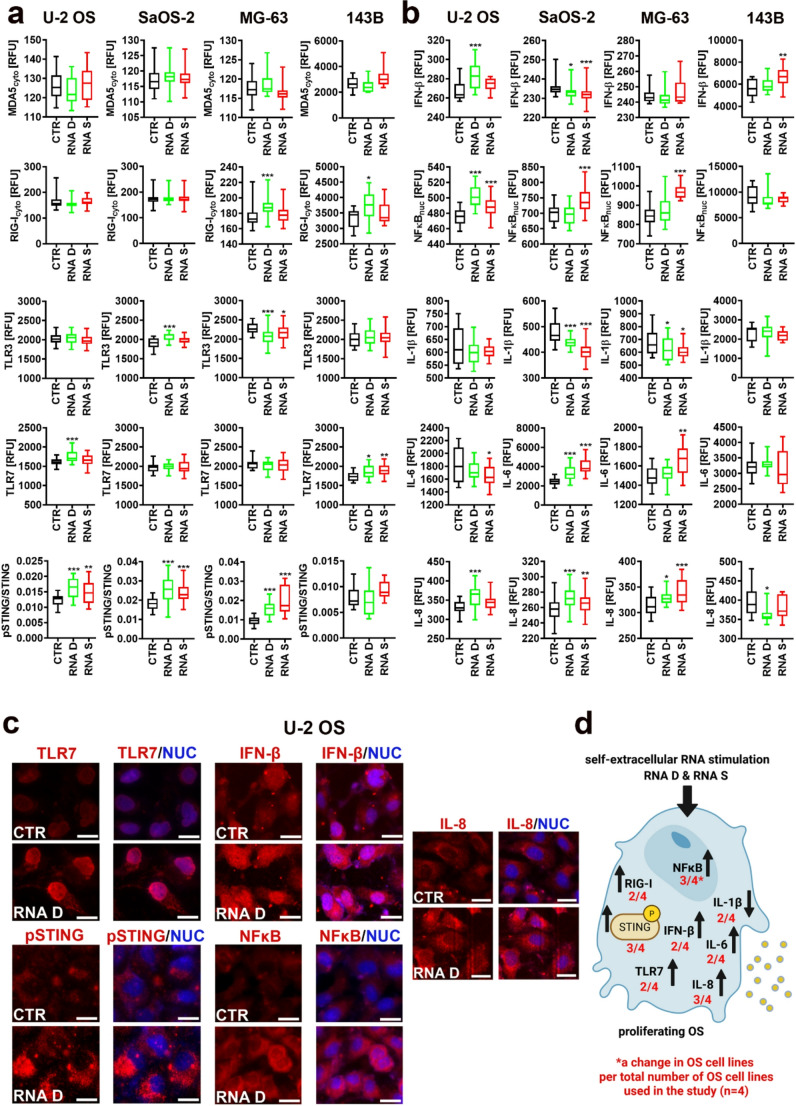
Self-extracellular RNA (RNA D and RNA S)-mediated changes in selected components of nucleic acid sensing pathways in proliferating OS cells. **a, b** Protein levels were analyzed using dedicated antibodies, immunofluorescence protocol, and imaging cytometry. Protein levels (cytosolic, nuclear or endosomal pools) are presented in relative fluorescence units (RFU). STING activity was assayed as a ratio of phosphorylated STING signals (foci) to STING signals. Box and whisker plots are shown, *n* = 3 (three biological replicates), ^***^p < 0.001, ^**^p < 0.01, ^*^p < 0.05 compared to untreated control (CTR) (ANOVA and Dunnett’s a posteriori test). RNA D, RNA released from dying OS cells; RNA S, RNA released from drug-induced senescent OS cells. **c** Representative microphotographs of imaging cytometry-based analysis of the levels of TLR7, pSTING, IFN-β, NFκB, and IL-8 (red) in U-2 OS cells in control (CTR) and treated conditions (RNA D treatment). Nuclei were visualized using Hoechst 33342 staining (blue). TLR7 signals were analyzed only in the endosomal compartment. TLR7 signals were also observed in the nucleus in U-2 OS cell line, but these signals were omitted from the analysis. Different patterns of TLR7 immunostaining in different OS cell lines were documented in the Supplementary Material. **d** A summarizing scheme showing the results of analyzed parameters (**a, b**) in OS cells. Increases and decreases are denoted using arrows. A change in analyzed parameter is also highlighted per total number of OS cell lines (*n* = 4, in red)

We have then analyzed if RNA D and RNA S treatment may affect the pools of two cytosolic RNA sensors, namely MDA5 and RIG-I [1, 2, 5, 34] in OS cells (Fig. 3a). RNA D caused an increase in the levels of RIG-I in MG-63 and 143B cells, whereas no MDA5-mediated response was observed (Fig. 3a). As limited cytosolic RNA sensor-based response was observed, the levels of two endosomal receptors TLR3 and TLR7 involved in dsRNA and ssRNA sensing and recognition, respectively [1–3], were then studied (Fig. 3a). The levels of TLR3 were elevated in RNA D-treated SaOS-2 cells, whereas increased pools of TLR7 were noticed upon RNA D stimulation in U-2 OS cells and RNA D and RNA S treatment in 143B cells (Fig. 3a). Surprisingly, RNA D and RNA S stimulation promoted the activity of STING, a component of the cGAS-cGAMP-STING DNA sensing pathway [2, 4] in three OS cell lines (Fig. 3a). There is evidence that downstream signaling elements of RNA and DNA sensing pathways can be physically and functionally interconnected [35–37]. For example, cGAS and STING proteins, responsible for the maintenance and DNA sensing, are also involved in the direct response to RNA viral infection [36]. The presence of DNA and RNA viruses may result in the upregulation of *RIG-I* and *STING* mRNA, respectively, assembly of the RIG-I/MAVS/STING complex and enhancement of interferon-based response [35]. However, increased phosphorylated signals of STING were accompanied by increased interferon β (IFN-β) production only in RNA D-treated U-2 OS cells (Fig. 3a-c). RNA S also caused an increase in the levels of IFN-β in 143B cells, but without a parallel increase in STING activity (Fig. 3a and b). Perhaps in RNA S-treated 143B cells, increased production of IFN-β may be a result of TLR7-mediated response (Fig. 3a and b). Furthermore, beside STING, TLR7 may also contribute to increased pools of IFN-β in U-2 OS cells subjected to RNA D treatment (Fig. 3a and b). As nucleic acid-induced activation of RIG-I, TLRs, and STING pathways may also promote NFκB-mediated production of proinflammatory cytokines [1–3], nuclear levels of NFκB were then analyzed as a sign of RNA D and/or RNA S-mediated NFκB activation (Fig. 3b). Indeed, RNA S induced the activity of NFκB as judged by increased nuclear signals of NFκB in three OS cell lines (Fig. 3b and d). Furthermore, RNA D also promoted the activity of NFκB in U-2 OS cells (Fig. 3b and c). RNA S-mediated increase in NFκB activity was accompanied by increased production of IL-6 and IL-8 in SaOS-2 and MG-63 cells (Fig. 3b). Similarly, RNA D-mediated increase in NFκB activity was correlated with increased levels of IL-8 in U-2 OS cells (Fig. 3b and c). One can conclude that different nucleic acid sensors can be activated upon RNA D and RNA S stimulation in phenotypically diverse OS cells contributing to IFN-β (143B cells) or NFκB-mediated proinflammatory response (SaOS-2 and MG-63 cells) or both responses (U-2 OS) (Fig. 3).

### m^5^C RNA methyltransferase-mediated response in self-extracellular RNA-stimulated OS cells

**Fig. 4:**
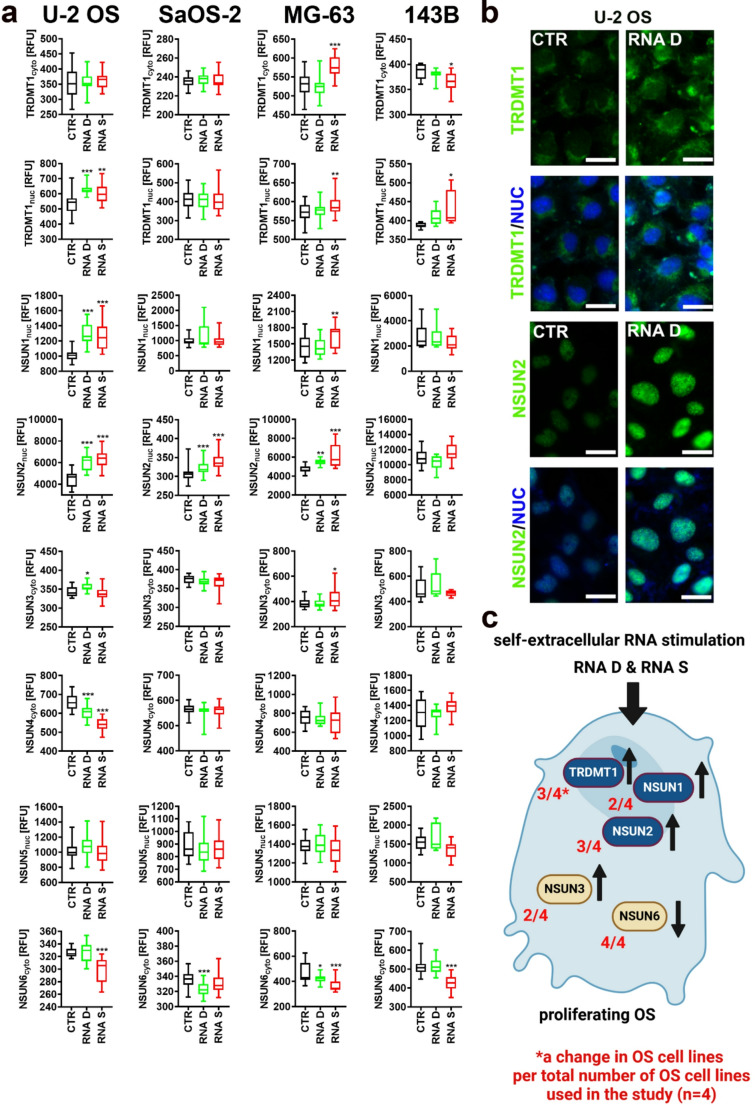
Self-extracellular RNA (RNA D and RNA S)-mediated changes in the levels of RNA m^5^C methyltransferases (TRDMT1 and NSUN proteins) in proliferating OS cells. **a** Protein levels were analyzed using dedicated antibodies, immunofluorescence protocol, and imaging cytometry. Protein levels (cytosolic or nuclear pools) are presented in relative fluorescence units (RFU). Box and whisker plots are shown, *n* = 3 (three biological replicates), ^***^p< 0.001, ^**^p < 0.01, ^*^p < 0.05 compared to untreated control (CTR) (ANOVA and Dunnett’s a posteriori test). RNA D, RNA released from dying OS cells; RNA S, RNA released from drug-induced senescent OS cells. **b** Representative microphotographs of imaging cytometry-based analysis of the levels of TRDMT1 and NSUN2 (green) in U-2 OS cells in control (CTR) and treated conditions (RNA D treatment). Nuclei were visualized using Hoechst 33342 staining (blue). **c** A summarizing scheme showing the results of analyzed parameters (**a**) in OS cells. Increases and decreases are denoted using arrows. A change in analyzed parameter is also highlighted per total number of OS cell lines (*n* = 4, in red)

As m^5^C RNA methyltransferases (m^5^C-RMT), both the NOL1/NOP2/sun (NSUN) family members and tRNA methyltransferase 1 TRDMT1, may modify foreign and host RNA molecules to modulate cellular function in normal and cancer cells [38–41], we decided then to study if NSUN1-6 and TRDMT1 may also be involved in RNA D and RNA S-induced response in OS cells (Fig. 4). Indeed, RNA S treatment resulted in an increase in the nuclear fraction of TRDMT1 in three OS cell lines (Fig. 4a). Furthermore, both RNA D and RNA S increased the levels of nuclear NSUN2 (Fig. 4a). Except for the RNA-mediated decrease in the levels of NSUN6 in four OS cell lines, the effects of other NSUN family members were less evident (Fig. 4a). TRDMT1 m^5^C-RMT (former name DNMT2), a modulator of longevity and different stress responses, namely oxidative, proteotoxic and DNA damage response (DDR), has been proposed as a novel target in anticancer therapy [41–43]. The status of *TRDMT1* gene, elevated and/or mutated in a number of cancer types, can be associated with cancer progression and therapeutic outcome [41]. For example, knockout of *TRDMT1* gene, a writer of RNA m^5^C at sites of DNA damage, sensitized OS cells to PARP inhibitors [42]. *TRDMT1* KO also promoted cellular heterogeneity of OS cell populations upon etoposide, DNA damaging agent, treatment and long-term culture (up to 28 days) as judged by affected DDR, telomere length, and retrotransposon activity [44]. This effect was also modulated by the stimulation with exogenous RNA derived from leukemia cells [44]. *TRDMT1* KO also sensitized drug-induced senescent OS cells to treatment with azacytidine, a hypomethylating agent, exerting a senolytic effect [12]. However, in selected experimental settings and cancer cell types, *TRDMT1* KO may also promote drug resistance, thus the effects of *TRDMT1* KO in cancer cells might depend on cellular context [41]. NSUN2 m^5^C-RMT has been also implicated in tumorigenesis of different cancer types [45]. NSUN2 is highly expressed in OS tissues and cells, and is associated with poor prognosis of OS patients [46]. Mechanistically, NSUN2 may promote progression of OS cells by methylation-based stabilization of fatty acid-binding protein 5 (*FABP5*) mRNA, stimulating fatty acid metabolism and cell proliferation [46].

### *TRDMT1* KO prevents STING activation and attenuates cell death signaling in RNA-treated senescent OS cells

**Fig. 5:**
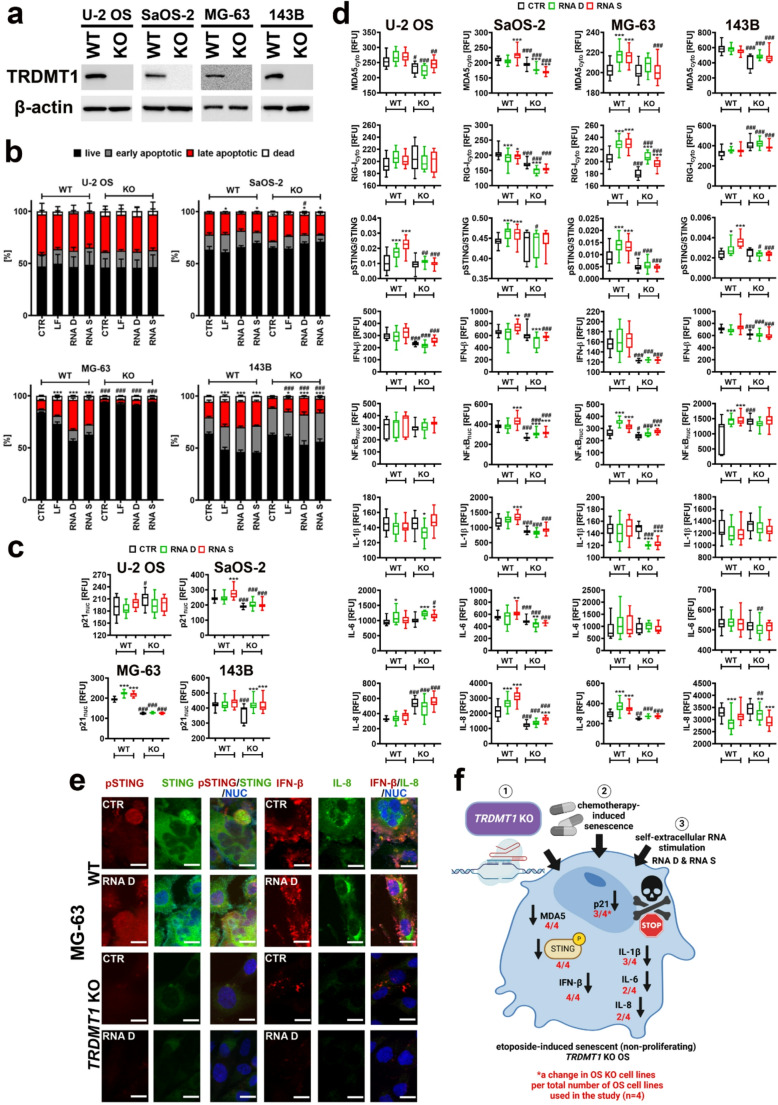
Self-extracellular RNA (RNA D and RNA S)-mediated cell death signaling and nucleic acid sensing in etoposide-induced senescent OS cells with *TRDMT1* KO. **a** *TRDMT1* KO in four OS cell lines was achieved using CRISPR/Cas9 technology and confirmed using western blotting. Representative immunoblots are shown. Beta-actin antibody served as a loading control. **b** Apoptosis was studied using Annexin V staining and flow cytometry. Lipofection control (LF) was also considered. Bars indicate SD, *n* = 3 (three biological replicates), ^***^p < 0.001, ^*^p< 0.05 compared to untreated control (CTR) (ANOVA and Dunnett’s a posteriori test), ^###^*p* < 0.001, ^#^*p* < 0.05 compared to corresponding WT sample (ANOVA and Tukey’s a posteriori test). **c** The analysis of the levels of p21, a cell cycle inhibitor and cell death mediator using anti-p21 antibody and imaging cytometry. Nuclear signals of p21 are presented in relative fluorescence units (RFU). **d** Nucleic acid sensing-related protein levels were analyzed using dedicated antibodies, immunofluorescence protocol, and imaging cytometry. Protein levels (cytosolic or nuclear pools) are presented in relative fluorescence units (RFU). STING activity was assayed as a ratio of phosphorylated STING signals (foci) to STING signals. Box and whisker plots are shown, *n* = 3 (three biological replicates), ^***^p < 0.001, ^**^p < 0.01, ^*^p < 0.05 compared to untreated control (CTR) (ANOVA and Dunnett’s a posteriori test), ^###^*p* < 0.001, ^##^*p*< 0.01, ^#^*p* < 0.05 compared to corresponding WT sample (ANOVA and Tukey’s a posteriori test). RNA D, RNA released from dying OS cells; RNA S, RNA released from drug-induced senescent OS cells; WT, control cells containing control plasmids; KO, CRISPR/Cas9-based *TRDMT1* KO cells. **e** Representative microphotographs of imaging cytometry-based analysis of the levels of pSTING and IFN-β (red), and STING and IL-8 (green) in WT and *TRDMT1* KO MG-63 cells in control (CTR) and treated conditions (RNA D treatment). Nuclei were visualized using Hoechst 33342 staining (blue). **f** A summarizing scheme showing the results of analyzed parameters (**b-d**) in OS cells. Decreases are denoted using arrows. A change in analyzed parameter is also highlighted per total number of OS cell lines (*n* = 4, in red)

As TRDMT1 nuclear fraction was upregulated upon RNA S stimulation in three OS cell lines (Fig. 4a), we decided then to analyze the effects of *TRDMT1* KO on RNA-related response in etoposide-induced senescent OS cells (Fig. 5). CRISPR/Cas9-mediated *TRDMT1* KO was confirmed using western blotting in four OS cell lines (Fig. 5a). In *TRDMT1* KO senescent OS cells (SaOS-2 and 143B cells), RNA-mediated apoptotic cell death was less pronounced than in corresponding WT cells (Fig. 5b). Furthermore, in the case of RNA-treated *TRDMT1* KO MG-63 cells, apoptosis was severely inhibited (Fig. 5b). Thus, in three senescent OS cell lines, *TRDMT1* KO resulted in more or less accented resistance to RNA D and RNA S treatment (Fig. 5b). In U-2 OS cells, RNA-mediated response was the most variable and no statistically differences in apoptotic marker were observed (Fig. 5b). In contrast to less accented apoptosis in WT proliferating metastatic cells (143B cells) compared to non-metastatic ones upon self-extracellular RNA stimulation, WT senescent 143B cells were not resistant to RNA D and RNA S treatment (Figs. 2c and 5b). Thus, the metastatic potential of OS cells may not solely determine the response to self-extracellular RNA as RNA-mediated effects may be modulated by proliferation status of OS cells, namely different responses may be observed in proliferating and non-proliferating (senescent) OS cells. Attenuated response to RNA treatment in *TRDMT1* KO may be associated with lowered levels of p21, a cell cycle inhibitor and cell death mediator [47, 48] compared to WT cells (Fig. 5c). This observation was the most accented in *TRDMT1* KO MG-63 cells characterized by the resistance to RNA stimulation (Fig. 5b and c). The effect of *TRDMT1* KO on nucleic acid sensing pathways was then also investigated (Fig. 5d). In senescent WT OS cells, RNA D and RNA S treatment caused activation of STING and this activation was prevented in corresponding *TRDMT1* KO in all four cellular models used (Fig. 5d and e). The levels of MDA5 and IFN-β were also lowered in *TRDMT1* KO compared to corresponding WT cells (Fig. 5d). RIG-I and NFκB-based responses were rather complex and no common features were observed in four OS cell lines examined (Fig. 5d). Except for U-2 OS cells, *TRDMT1* KO resulted in a decrease in the pools of proinflammatory cytokines IL-6 and IL-8 (Fig. 5d). In conclusion, limited cell death response to RNA D and RNA S treatment in senescent *TRDMT1* KO OS cells may be associated with decreased p21 levels and the attenuation of STING activity and related proinflammatory cytokine secretion (Fig. 5f). For example, p21 overexpression promoted apoptosis in glioblastoma cell lines, and in remaining (surviving) cells, cell cycle arrest and activation of senescence program were observed [48]. The activation of cGAS-STING signaling in cancer may be considered as a double-edged sword phenomenon as both STING-mediated tumor suppressive and tumor promoting effects may be noted [49, 50]. In cancer cells with elevated cytosolic pools of dsDNA due to e.g., cancer-associated genomic instability, the attenuation of STING-based signaling may protect against immune infiltration and related cell death [49]. For example, forced activation of the STING/IRF3/IFN-β pathway by the use of sodium-glucose cotransporter 2 (SGLT2) inhibitor resulted in immune infiltration-based antitumor effects in OS cells that was even potentiated when OS cells were co-treated with a STING agonist 2’,3’-cyclic GMP-AMP (cGAMP) [51]. It is worth noting that etoposide-induced damaged nuclear DNA may also trigger PARP1 and ATM-mediated non-canonical activation of STING and IFN-β signaling promoting NFκB-dependent transcriptional program and an innate immune response [52]. cGAS-STING pathway was also reported as a crucial regulator of senescence and senescence-associated secretory phenotype (SASP) [53]. Cytoplasmic chromatin fragments, mtDNA and cDNA may trigger cGAS-STING-based production of proinflammatory cytokines in senescent cells and cGAS-STING pathway may be considered as a novel target during replicative senescence in normal cells and therapy-induced senescence in cancer cells [54]. RIG-I and MDA5 may also promote SASP in senescent cells with mitochondrial dsRNA presented in the cytosol and their inhibition may lower senescence-associated proinflammatory response [55]. In our experimental conditions, *TRDMT1* KO in RNA-treated drug induced-senescent OS cells also diminished STING activity, MDA5 levels, and related cytokine production, but this resulted in limited cell death signaling compared to WT cells (Fig. 5f). Thus, TRDMT1 activity might be needed for self-extracellular RNA-based response and the induction of cell death signals during chemotherapeutic treatment as shown using selected cellular models of OS in vitro with activated therapy-induced senescence.

### Identification of IFN-β binding RNA partners and the effect of *TRDMT1* KO

**Fig. 6:**
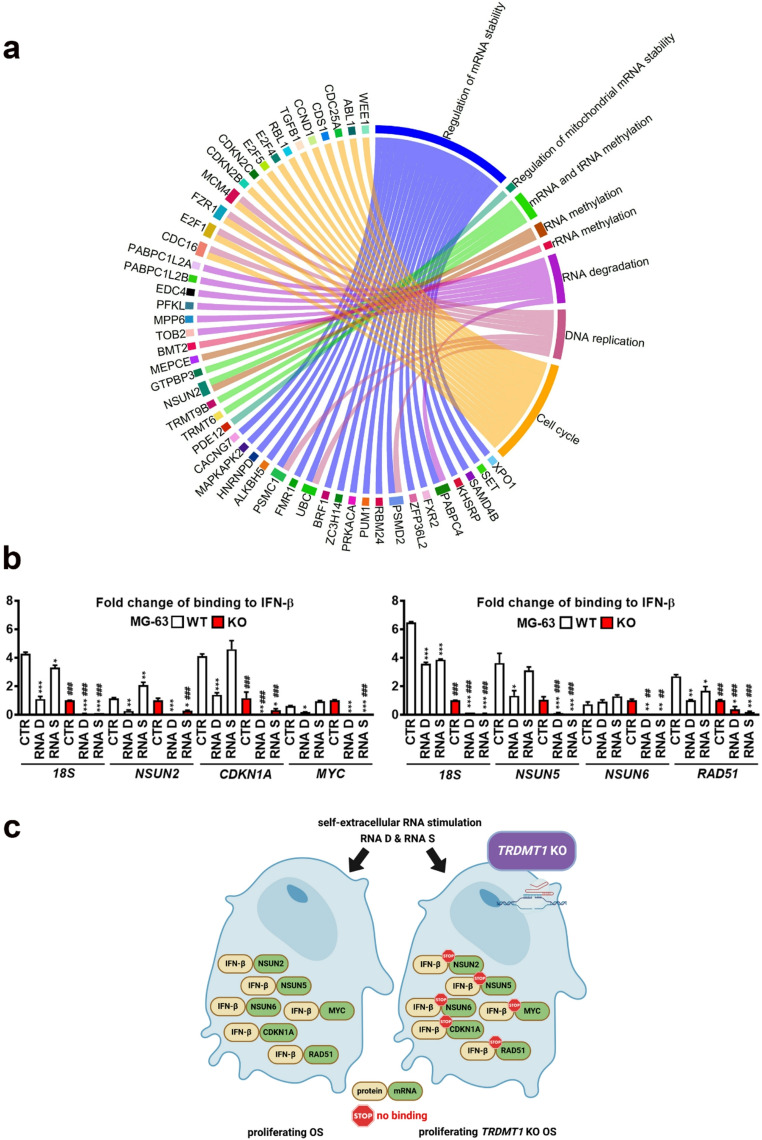
*In silico* analysis of interferon beta binding RNA partners (**a**) and the effect of *TRDMT1* KO and self-extracellular RNA (RNA D and RNA S) treatment on detected physical associations in MG-63 OS cell line (**b**). **a** Circos style plot representing predicted genes with high-confidence of protein-RNA interactions of interferon beta and their association with selected pathways of interest. A circos plot was generated using R software and circlize package. More information can be found in the Supplementary Material. **b** Experimental validation of selected interferon beta binding RNA partners, namely *NSUN2*, *NSUN5*, *NSUN6*, *CDKN1A*, *MYC*, and *RAD51* transcripts in OS cells using RIP assay. *18S* gene served as a housekeeping gene. Data are presented as a fold change of binding to IFN-β. Bars indicate SD, *n* = 3 (three biological replicates),^***^p < 0.001, ^**^p < 0.01, ^*^p< 0.05 compared to untreated control (CTR) (ANOVA and Dunnett’s a posteriori test), ^###^*p* < 0.001, ^##^*p* < 0.01 compared to corresponding WT sample (ANOVA and Tukey’s a posteriori test). RNA D, RNA released from dying OS cells; RNA S, RNA released from drug-induced senescent OS cells; WT, control cells containing control plasmids; KO, CRISPR/Cas9-based *TRDMT1* KO cells. **c** A summarizing scheme showing the effect on *TRDMT1* KO and self-extracellular RNA (RNA D and RNA S) treatment on IFN-β binding to selected transcripts in OS cells.

We have noticed that IFN-β can form some kind of clusters in etoposide-induced senescent OS cells both WT and *TRDMT1* KO cells, and IFN-β levels were decreased in *TRDMT1* KO cells compared to WT OS cells (Fig. 5d and e), and concluded that perhaps such IFN-β-based clusters may contain also non-protein biomolecules such as nucleic acids, e.g., RNA. We have then hypothesized that RNA can be considered as a scaffold to form such IFN-β-based clusters and decided to investigate putative RNA partners of IFN-β. First, in silico analysis was conducted and the transcripts involved in the regulation of RNA methylation, stability, and degradation, DNA replication, and cell cycle were revealed (Fig. 6a). As *NSUN2* transcript was identified as a potential RNA partner of IFN-β (Fig. 6a), the physical association of selected transcripts from NSUN family members, proliferation and DNA repair regulators with IFN-β was then studied using RIP assay (Fig. 6b). IFN-β binding with the following transcripts were documented, namely *NSUN2*, *NSUN5*, *NSUN6*, *CDKN1A* (p21), *MYC*, and *RAD51* in MG-63 cells (Fig. 6b). In general, *TRDMT1* KO diminished the physical association of *NSUN2*, *NSUN5*, *NSUN6*, *CDKN1A*, *MYC*, and *RAD51* with IFN-β (Fig. 6b). This effect was potentiated by RNA D and RNA S treatment (Fig. 6b and c). Thus, novel IFN-β binding partners were discovered with the possibility of methylation-based regulation of cellular function (NSUN proteins), proliferation, cell cycle arrest, and mode of cell death (p21) and DDR (RAD51). This regulation may rely on TRDMT1 activity and may be modulated by RNA-based responses (Fig. 6b and **c**). Our experimental system has some limitations. First of all, RIP assay does not discriminate between direct and indirect binding. Thus, more studies are needed to reveal the nature of IFN-β binding to detected transcripts. Second, a decrease in the pools of IFN-β in *TRDMT1* KO MG-63 cells (Fig. 5d) may contribute to observed limited binding of IFN-β to selected transcripts in *TRDMT1* KO MG-63 cells (Fig. 6b). Thus, more studies are needed to validate the importance of IFN-β binding to detected transcripts as well as the role of TRDMT1. It is widely accepted that interferon secretion activates interferon-based response in cells with interferon receptors leading to the expression of hundreds of interferon-stimulated genes (ISGs) and promoting antiviral defense, antiproliferative effects, and adaptive immunity [56]. Nevertheless, the role of type I interferon (IFN-I) in cancer biology and therapy is rather complex [57]. In cancer cells, acute exposure to high dose IFN-I resulted in the inhibition of cell proliferation and cell death, whereas chronic low dose treatment with IFN-I stimulated pro-survival response [57]. Aberrant DNA synthesis and DNA damage in cancer cells may promote IFN-β production and related expression of ISGs with anti-tumor effects (cell growth arrest, senescence, cell death) [57]. However, high persistent levels of unphosphorylated (U-) IFN-stimulated gene (ISG) factor 3 (ISGF3) (U- ISGF3), as a result of elevated levels of *STAT1*, *STAT2*, and *IRF9* transcripts, may also exert pro-tumor effects [57].

### *TRDMT1* KO affects replication fork dynamics in RNA-treated OS cells

**Fig. 7:**
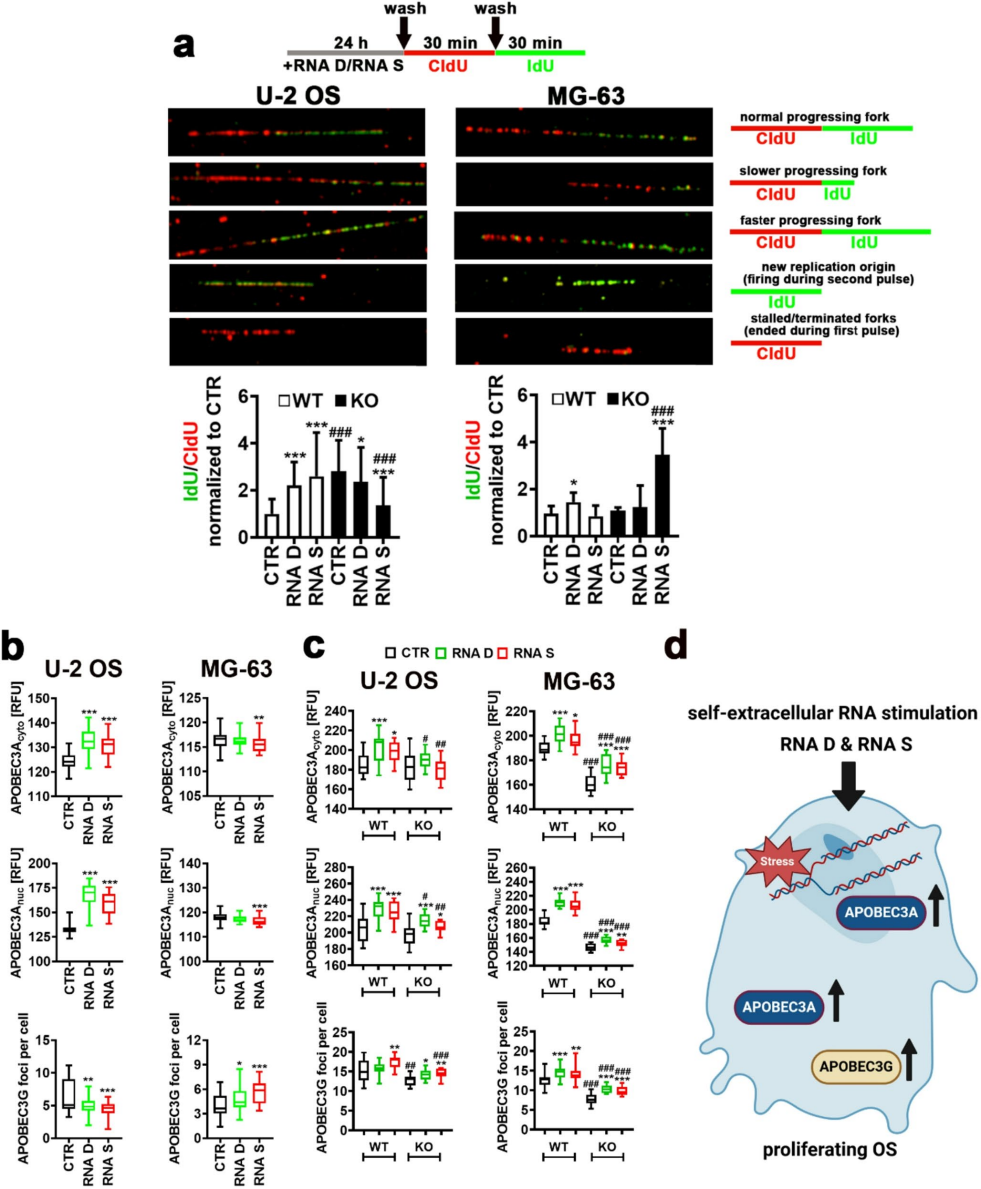
The effect of *TRDMT1* KO and self-extracellular RNA (RNA D and RNA S) treatment on replication fork dynamics (**a**) and the levels of cytosine deaminase family members APOBEC3A and APOBEC3G (**b, c**) in OS cells. **a** Perturbations in replication fork progression were studied using DNA fiber assay. Data are presented as a ratio of IdU (green) to CldU (red) normalized to CTR (WT). Representative microphotographs and schemes showing typical examples of progressing fork types are shown. Bars indicate SD, *n* = 3 (three biological replicates), ^***^p < 0.001, ^*^p< 0.05 compared to untreated control (CTR) (ANOVA and Dunnett’s a posteriori test), ^###^*p* < 0.001 compared to corresponding WT sample (ANOVA and Tukey’s a posteriori test). (**b**, proliferating OS cells; **c**, etoposide-induced senescent WT and *TRDMT1* KO OS cells). The levels of APOBEC3A and APOBEC3G were investigated using dedicated antibodies and imaging cytometry. In the case of APOBEC3A, cytosolic and nuclear levels are presented in relative fluorescence units (RFU). In the case of APOBEC3G, APOBEC3G foci were counted per cell. **b, c** Box and whisker plots are shown, *n*= 3 (three biological replicates), ^***^p < 0.001, ^**^p< 0.01, ^*^p < 0.05 compared to untreated control (CTR) (ANOVA and Dunnett’s a posteriori test. RNA D, RNA released from dying OS cells; RNA S, RNA released from drug-induced senescent OS cells. (**c**) ^###^*p*< 0.001, ^##^*p* < 0.01, ^#^*p* < 0.05 compared to corresponding WT sample (ANOVA and Tukey’s a posteriori test). WT, control cells containing control plasmids; KO, CRISPR/Cas9-based *TRDMT1* KO cells. **d** A summarizing scheme showing the results of analyzed parameters (**a** and **b**) in proliferating OS cells. Increases are denoted using arrows.

As TRDMT1 may take part in DNA damage response [42] and DNA replication may be affected in *TRDMT1* KO due to compromised binding of IFN-β with transcripts regulating DNA synthesis (Fig. 6a), the progression of DNA replication and its potential perturbations by *TRDMT1* KO and RNA D and RNA S treatment were then investigated using DNA fiber assay (Fig. 7a). In RNA-treated WT OS cells, replication dynamics was accelerated as judged by increased ratio of IdU signals to CldU signals (Fig. 7a). Similar effect was observed in *TRDMT1* KO U-2 OS cells without any treatment (Fig. 7a). In *TRDMT1* KO MG-63 cells, RNA S treatment also significantly potentiated replication progression compared to RNA S-treated WT corresponding cells and untreated *TRDMT1* KO cells (Fig. 7a). In contrast, RNA D and RNA S treatment in *TRDMT1* KO U-2 OS cells decreased the progression of DNA replication (Fig. 7a) that may be considered as a sign of replication stress with potential implications to genome stability and cell survival [58]. Indeed, etoposide-treated *TRDMT1* KO U-2 OS cells were more susceptible to DNA breaks formation compared to corresponding WT cells that was also accompanied by attenuated DDR as judged by decreased phosphorylation status of ATM and H2AX, two components of DDR [44]. It was also reported that affected replication fork protection may be accompanied by cGAS-STING-dependent innate immune signaling [59] and STING activity may contribute to nascent DNA degradation [60]. In unperturbed cells, STING depletion also inhibited fork progression rate [60]. Perhaps affected replication fork progression in RNA-treated OS cells (Fig. 7a) may be also associated with elevated STING activity in our experimental settings (Fig. 3a).

Apolipoprotein B mRNA-editing enzyme catalytic polypeptide-like 3 (APOBEC3) family of enzymes, involved in cytidine deaminase-mediated antiviral responses, are also implicated in the stimulation of uracil-induced somatic mutational heterogeneity and chromosomal instability in cancer cells that might be potentiated if APOBEC3 proteins are overexpressed, mutated, and localized in the nucleus or when replication stress is promoted [61–65]. Thus, we decided next to investigate if two members of APOBEC3 family, namely APOBEC3A and APOBEC3G might also be associated with RNA-mediated perturbations in replication fork dynamics (Fig. 7b). In RNA-treated U-2 OS and MG-63 cells, accelerated replication fork progression was accompanied by elevated levels of APOBEC3A and APOBEC3G, respectively (Fig. 7b). As APOBEC3 expression was also augmented during replication stress-associated senescence [65], we also analyzed the levels of APOBEC3A and APOBEC3G in *TRDMT1* KO OS cells in a non-proliferating state (drug-induced senescence) and their modulation by RNA stimulation (Fig. 7c). RNA treatment also caused an increase in the levels of APOBEC3A and APOBEC3G in WT senescent OS cells (Fig. 7c). In *TRDMT1* KO senescent OS cells, similar observation was noticed, but, in general, the levels of APOBEC3A and APOBEC3G were lowered compared to corresponding WT senescent OS cells (Fig. 7c). This effect can be due to lower levels of IFN-β in *TRDMT1* KO senescent OS cells compared to corresponding WT cells (Fig. 5d), and interferon-mediated stimulation of APOBEC expression in different cellular systems [66, 67]. In conclusion, in proliferating OS cells, RNA treatment and *TRDMT1* KO affected replication fork dynamics (Fig. 7d). In selected experimental settings, perturbed replication progression and increased levels of APOBEC3A and APOBEC3G were observed (Fig. 7d). However, more studies are needed to document the importance of APOBEC3A and APOBEC3G-mediated response in WT and *TRDMT1* KO senescent cells (non-proliferating cells with inhibited DNA replication).

**Fig. 8:**
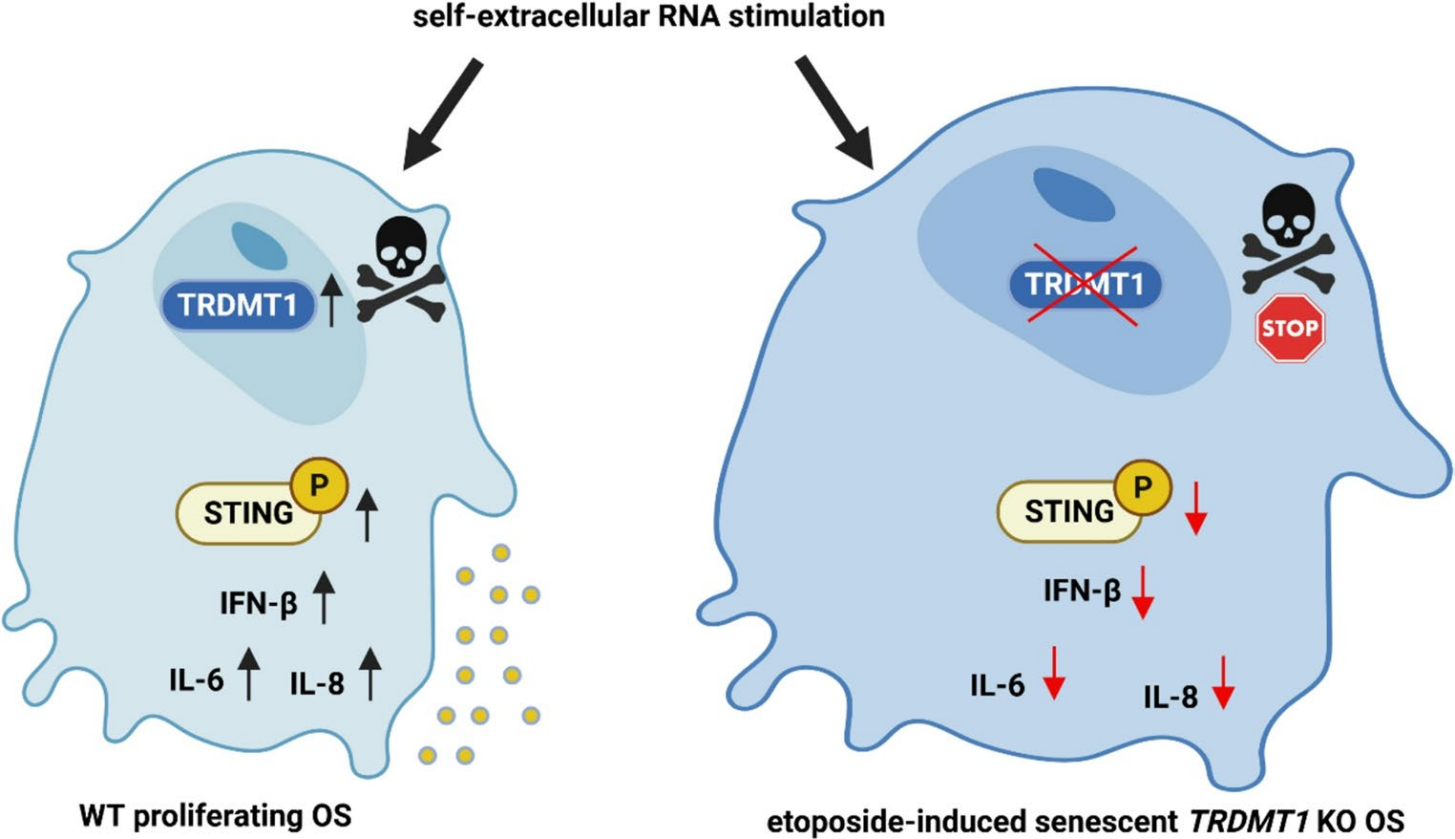
A summarizing scheme showing the role of TRDMT1 methyltransferase during self-extracellular RNA (RNA D or RNA S)-mediated response in OS cells. Opposite changes in the levels of selected parameters in self-extracellular RNA-treated proliferating (left) and drug-induced senescent (right) OS cells with *TRDMT1* KO are denoted. Upon stimulation with self-extracellular RNA (RNA D or RNA S), nuclear pools of TRDMT1 were increased that was accompanied by STING activation and related proinflammatory response and cell death (black arrows). In contrast, treatment with self-extracellular RNA of non-proliferating OS cells with *TRDMT1* KO (etoposide-induced model of cellular senescence) resulted in attenuated STING-based response, decreased secretion of proinflammatory factors, and limited cell death signaling (red arrows). IFN-β, interferon beta; IL-6, interleukin-6; IL-8, interleukin-8

In conclusion, we show that RNA released from dying or etoposide-induced senescent OS cells reduced cell survival, promoted nitro-oxidative stress, nucleic acid sensing, and TRDMT1 and NSUN2-based responses in proliferating OS cells (Figs. 2, 3 and 4). RNA treatment also perturbed replication fork dynamics and caused an elevation in the levels of APOBECs (Fig. 7). *TRDMT1* KO in RNA-treated drug-induced senescent OS cells resulted in diminished STING activity, cytokine secretion, and cell death signaling (Fig. 5). RNA-mediated opposite effects on the pools of selected parameters that were observed in proliferating versus etoposide-induced senescent OS with *TRDMT1* KO are highlighted in Fig. 8. We postulate that TRDMT1 activity might be important, at least in OS cells, during chemotherapy-induced cell death and senescence accompanied by the release of self-extracellular RNA and the induction of related nuclei acid signaling pathways.

## Supplementary information

Below is the link to the electronic supplementary material.

ESM 1 (XSLX 2.10 MB)

ESM 2 (XSLX 80.0 KB)

ESM 3 (DOCX 5.88 KB)

ESM 4 (DOCX 3.76 KB)

ESM 5 (XSLX 821 KB)

ESM 6 (DOCX 1.36 MB)

## Data Availability

All data are available from the corresponding author upon reasonable request and in the Supplementary Material (supplementary information to NGS analysis presented in Fig. 1b-d, TLR7 immunostaining presented in Fig. 3c, and in silico analysis presented in Fig. 6a). NGS data were deposited at the GEO repository (GEO accession number GSE296519).
