# Supplementary material for: TRDMT1 methyltransferase gene knockout attenuates STING-based cell death signaling during self-extracellular RNA-mediated response in drug-induced senescent osteosarcoma cells
Source: Cell Mol Life Sci. 2025 Aug 13;82(1):310. doi: 10.1007/s00018-025-05835-1 (PMC12350886; doi:10.1007/s00018-025-05835-1)
Supplement: Supplementary file 6 — (DOCX 1.36 MB) [file 18_2025_5835_MOESM6_ESM.docx]

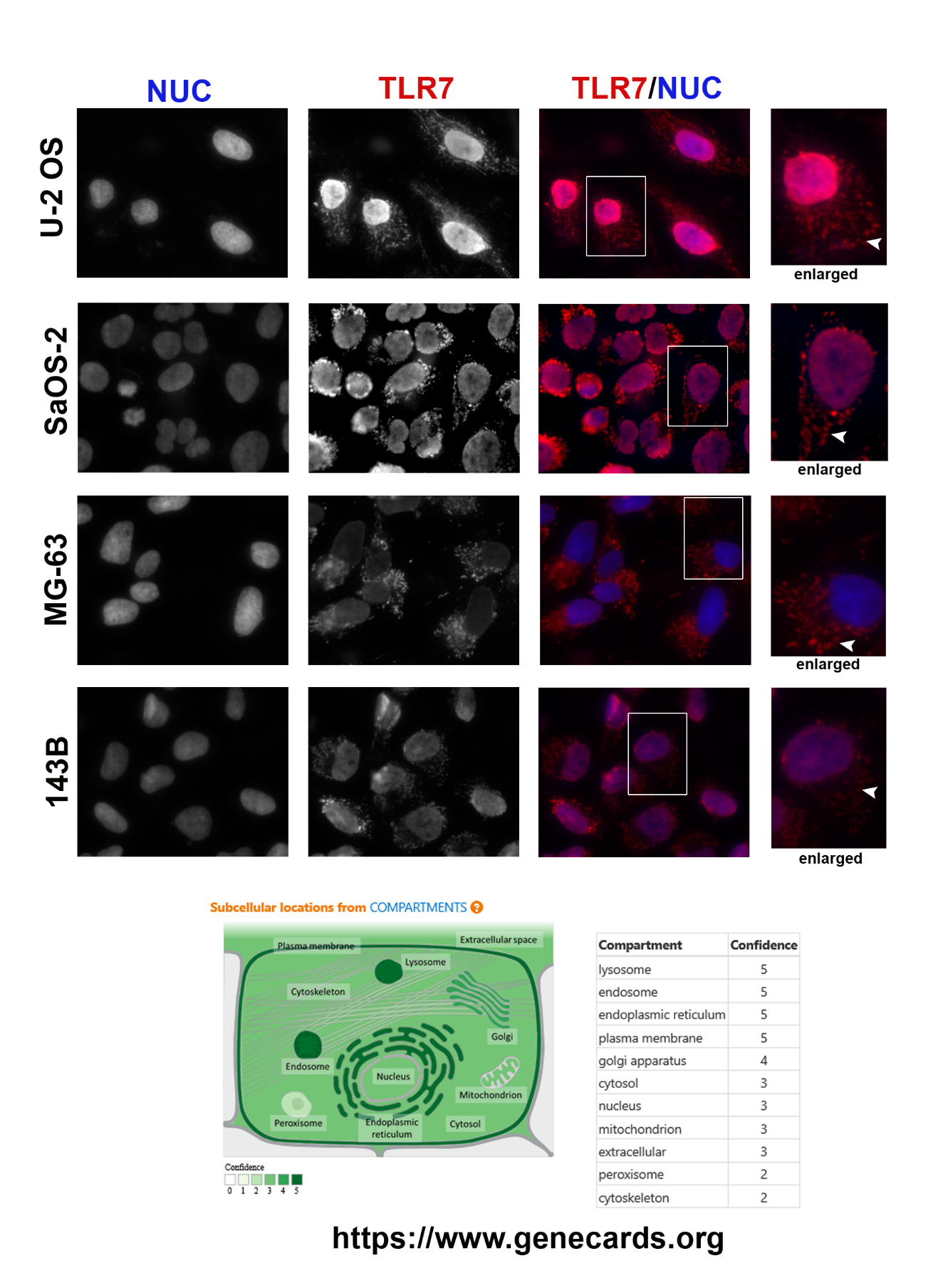


**Supplementary Figure 1.** Representative microphotographs of imaging cytometry-based analysis of the levels of TLR7 in all four OS cell lines used (top, red). Enlarged microphotographs are denoted in a white frame. Arrowheads show TLR7 signals in endosomal compartment that were analyzed. For some cell lines, e.g., U-2 OS cells, TLR7 signals were also observed in the nucleus that may rely on TLR7 signals in endoplasmic reticulum adjacent to the nucleus. Nuclei were visualized using Hoechst 33342 staining (NUC, blue). Monochromatic microphotographs are also shown. Information on subcellular locations of TLR7 is also presented based on data available at [www.genecards.org](http://www.genecards.org) (bottom).
